# Incidence, Impact, and Predictors of Cranial Nerve Palsy and Haematoma Following Carotid Endarterectomy in the International Carotid Stenting Study

**DOI:** 10.1016/j.ejvs.2014.08.002

**Published:** 2014-11

**Authors:** D. Doig, E.L. Turner, J. Dobson, R.L. Featherstone, G.J. de Borst, M.M. Brown, T. Richards, the ICSS Investigators

**Affiliations:** a Institute of Neurology, University College London, London, UK; b Department of Biostatistics and Bioinformatics and Duke Global Health Institute, Duke University, Durham, NC, USA; c Department of Medical Statistics, London School of Hygiene and Tropical Medicine, London, UK; d Department of Vascular Surgery, University Medical Centre Utrecht, Utrecht, The Netherlands; e Department of Surgical and Interventional Sciences, University College London, London, UK

**Keywords:** Carotid atherosclerosis, Carotid artery stenosis, Carotid endarterectomy, Cranial nerves, Haematoma

## Abstract

**Objective:**

Cranial nerve palsy (CNP) and neck haematoma are complications of carotid endarterectomy (CEA). The effects of patient factors and surgical technique were analysed on the risk, and impact on disability, of CNP or haematoma in the surgical arm of the International Carotid Stenting Study (ICSS), a randomized controlled clinical trial of stenting versus CEA in patients with symptomatic carotid stenosis.

**Materials and methods:**

A per-protocol analysis of early outcome in patients receiving CEA in ICSS is reported. Haematoma was defined by the surgeon. CNP was confirmed by an independent neurologist. Factors associated with the risk of CNP and haematoma were investigated in a binomial regression analysis.

**Results:**

Of the patients undergoing CEA, 45/821 (5.5%) developed CNP, one of which was disabling (modified Rankin score = 3 at 1 month). Twenty-eight (3.4%) developed severe haematoma. Twelve patients with haematoma also had CNP, a significant association (*p* < .01). Independent risk factors modifying the risk of CNP were cardiac failure (risk ratio [RR] 2.66, 95% CI 1.11 to 6.40), female sex (RR 1.80, 95% CI 1.02 to 3.20), the degree of contralateral carotid stenosis, and time from randomization to treatment >14 days (RR 3.33, 95% CI 1.05 to 10.57). The risk of haematoma was increased in women, by the prescription of anticoagulant drugs pre-procedure and in patients with atrial fibrillation, and was decreased in patients in whom a shunt was used and in those with a higher baseline cholesterol level.

**Conclusions:**

CNP remains relatively common after CEA, but is rarely disabling. Women should be warned about an increased risk. Attention to haemostasis might reduce the incidence of CNP. ICSS is a registered clinical trial: ISRCTN 25337470.

**What this paper adds**

Perioperative cranial nerve palsy remains an important complication of carotid endarterectomy, and occurred in 5.5% of patients in a recent large randomized trial. There is a significant association between the occurrence of perioperative haematoma and perioperative cranial nerve palsy. Patients can be reassured that many cranial nerve palsies following carotid endarterectomy are transient and non-disabling. Women should be warned about an increased risk of cranial nerve palsy.

## Introduction

Evidence from randomized trials of medical therapy versus carotid endarterectomy (CEA) for symptomatic stenosis of the carotid artery^1^ has led to the recommendation that CEA should be performed in patients with symptomatic carotid artery stenosis to reduce the long-term risk of recurrent stroke or TIA.^2^ The combined rate of stroke or death at 30 days following CEA in NASCET, ECST, and the VA trials was 7.1% (95% CI 6.3 to 8.1%).^3^ However, the primary endpoints of these trials did not include cranial nerve palsy (CNP) or haematoma. Although less extensively studied, the surgical complications of CNP and haematoma have long been recognized following CEA,^4^ and have been associated with an increased risk of stroke or death.^5^ Nerves affected include the mandibular branch of the facial nerve, vagal, glossopharyngeal, hypoglossal, and accessory nerves,^4^, ^6^, ^7^ and therefore CNP has the potential to cause significant postoperative morbidity.

Carotid angioplasty and stenting (CAS) was developed as an alternative to CEA, in part to avoid these hazards of a surgical incision. However, the results of recent large randomized trials, including the International Carotid Stenting Study (ICSS),^8^ have consistently shown that CAS carries a higher risk of non-disabling stroke than CEA within 30 days of the procedure, with no significant difference in the rates of disabling stroke or death.^9^ ICSS was a randomized controlled multicentre open clinical trial that randomized patients with symptomatic carotid stenosis to CEA or CAS. In this study, the incidence and severity of CNP and haematoma in ICSS, and risk factors for their development, were studied, to identify groups of patients at higher risk and determine whether these complications merit consideration in selection of a revascularization procedure.

## Methods

### Study design

#### Patient selection and protocol design

The protocol for ICSS is published elsewhere.^10^ Patients over 40 years old were eligible for randomization in ICSS if they had more than 50% recently symptomatic carotid stenosis suitable for either CAS or CEA, and were clinically stable. Patients were excluded if they had a major stroke with poor recovery of function, if their vascular anatomy rendered CAS or CEA unsuitable, if the stenosis was caused by non-atheromatous disease, if cardiac bypass was planned within 1 month of the revascularization procedure, or if there had been previous revascularization of the symptomatic artery.

Carotid endarterectomy in ICSS was performed according to the surgeon's usual practice: local, general, or combined anaesthesia was allowed for the procedure. The type of arterial reconstruction to be carried out was not specified in the protocol, nor was the choice of peri-procedural medication.

#### Outcome events

Technical details of the surgical procedure and the occurrence of cranial nerve palsy or haematoma were reported by trial investigators. All patients were then re-assessed at 1 month after the procedure by a neurologist or investigator under their supervision. CNPs were adjudicated internally at the ICSS trial office and judged to be disabling if the patient's score on the modified Rankin Scale (mRS) increased to 3 or more at 30 days after the procedure, where that increase was attributable to the CNP. Investigators were additionally asked to complete a questionnaire (Appendix I) giving details of the clinical consequences of CNP and whether or not the lesion resolved during subsequent follow-up in the trial. Haematoma was classified as severe if it required re-operation, transfusion, or prolonged hospital stay.

#### Statistical analysis

The data were analysed per-protocol; only patients in whom the randomly allocated procedure was initiated were included in this analysis. Patients who crossed over or received CEA after an attempt at stenting were excluded. A procedure was deemed to have been initiated if the patient underwent either local or general anaesthesia prior to commencement of surgery. Risk factors for CNP and haematoma were examined sequentially in a univariable binomial regression analysis using maximum likelihood estimation. The risk ratio for each factor was estimated with a 95% confidence interval. Wald tests were used for continuous and binary predictors, with an overall likelihood ratio test for categorical predictors of more than two levels. A multivariable model was developed using a forward stepwise based approach. Patients with missing data were excluded from each relevant analysis. Analyses were performed with Stata (StataCorp. 2011. *Stata Statistical Software: Release 12*. College Station, TX: StataCorp LP).

## Results

### Cranial nerve palsy

In ICSS, 1713 patients were randomized. Of 858 patients randomized to CEA, the allocated procedure was initiated in 821 (95.7%). Four of 821 patients (0.5%) died between initiation of the procedure and 30 days post-procedure. Forty-five of 821 patients (5.5% of initiated CEAs) were reported to have CNP within 30 days of the procedure. The results of adjudication of which cranial nerves were affected are presented in Table 1. A total of 50 CNPs were reported: facial (*n* = 23), vagus (6), hypoglossal (13), glossopharyngeal (4), accessory (1), and trigeminal (1); in two patients it was not possible to determine which cranial nerve was affected. One CNP was judged to be disabling with an mRS of 3 at 1-month follow-up. This patient had glossopharyngeal nerve palsy with impairment in swallowing requiring placement of a naso-gastric feeding tube. Two cranial nerve palsies were reported in each of five trial participants. In those patients with CNP where symptomatic resolution was confirmed (*n* = 20), the median duration of symptoms before resolution was 30 days (minimum 2 days, maximum 520 days). The exact duration of symptoms in the remainder was undetermined. In only two of 821 patients (0.2%) were the symptoms reported to have not resolved during follow-up of 6.4 and 3.1 years, respectively. One of these patients experienced voice hoarseness caused by vocal cord paresis following vagal nerve injury. The other experienced uplifting of the mouth on one side as a result of facial nerve injury.

**Table 1:** Summary of cranial nerve palsies (CNPs) within 30 days of endarterectomy in ICSS per-protocol participants in whom the procedure was initiated (*n* = 821).

Cranial nerve	Number of CNPs (*n* = 50 in 45 patients)	Number of disabling CNPs (mRS ≥ 3 because of CNP)	Number of CNPs confirmed persisting after 30 days
Facial	23	0	4
Hypoglossal	13	0	2
Vagus	6	0	4
Accessory	1	0	0
Glossopharyngeal	4	1	1
Trigeminal	1	0	0
Undetermined	2	0	0

CNP = cranial nerve palsy; ICSS = International Carotid Stenting Study; mRS = modified Rankin Scale score.

**Figure 1:**
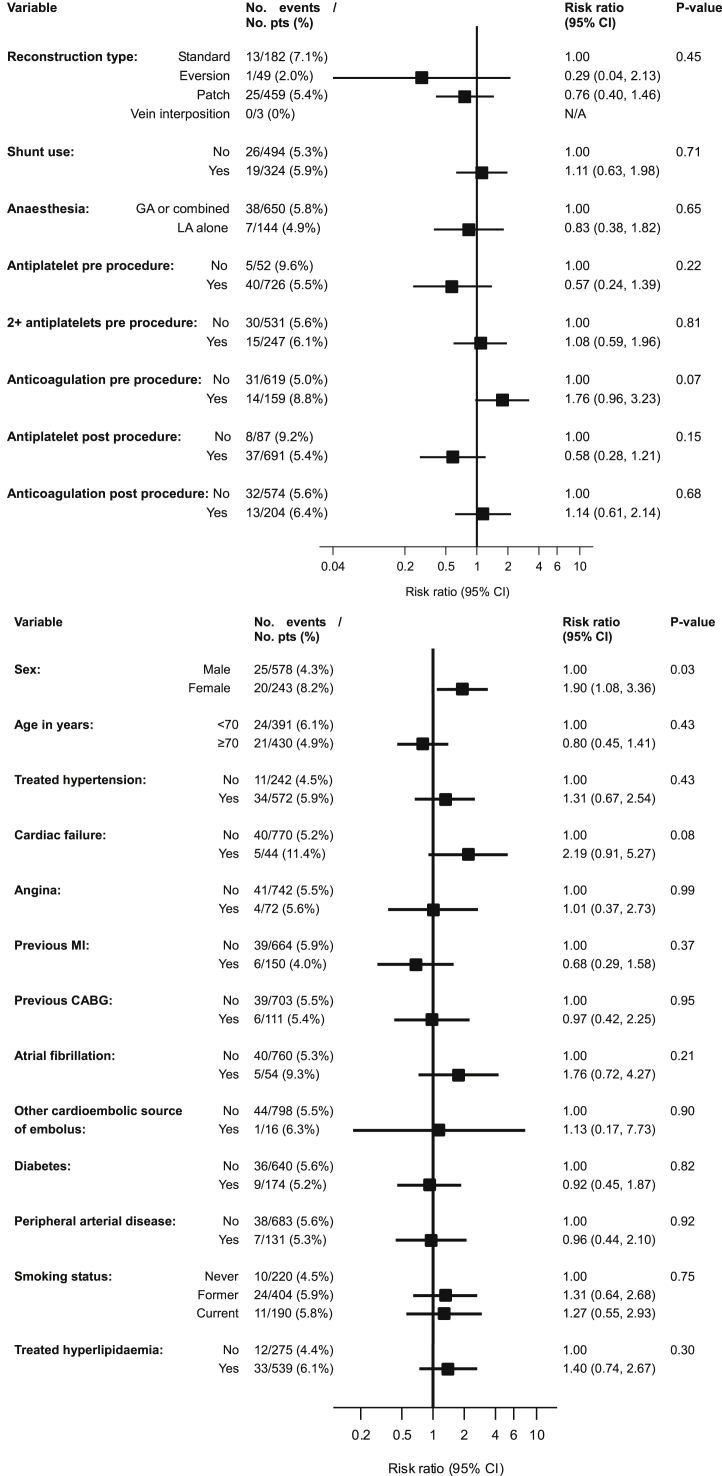

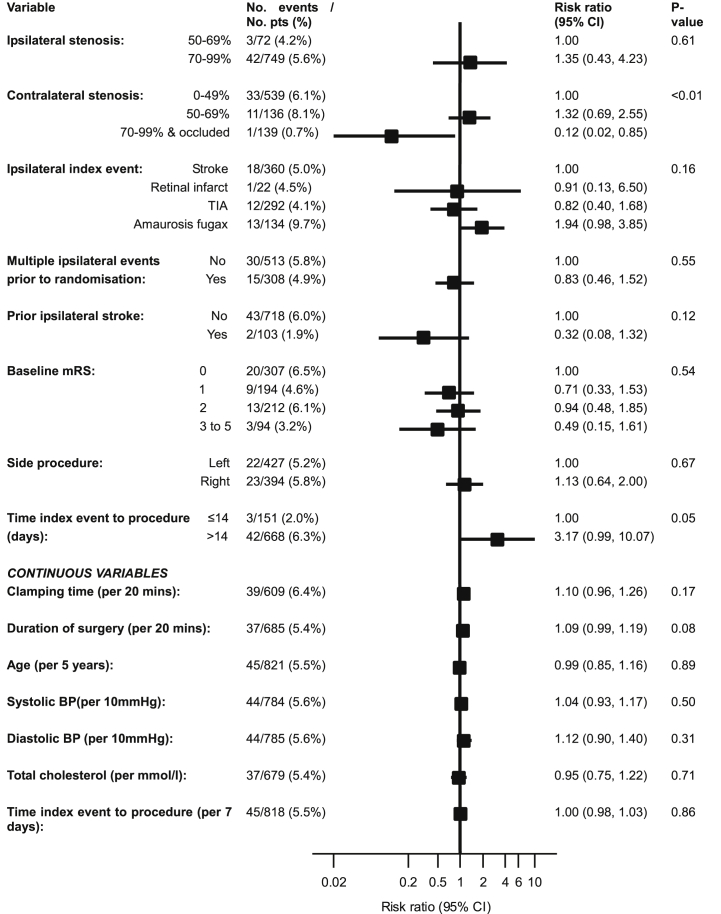
Univariable predictors of risk of cranial nerve palsy within 30 days of endarterectomy in ICSS per-protocol participants in whom the procedure was initiated (n=821).

The results of the risk factor analysis are presented in Fig. 1. Statistically significant predictors of CNP in univariable analysis were female sex (risk ratio [RR] 1.90, 95% CI 1.08 to 3.36, *p* = .03) and a high degree of contralateral carotid artery stenosis. Other demographic and technical factors, including the type of arterial reconstruction, type of anaesthesia or shunt use did not predict CNP. Independent predictors of CNP in multivariable analysis, summarised in Table 2, were cardiac failure (RR 2.66, 95% CI 1.11 to 6.40, *p* = .03), female sex (RR 1.80, 95% CI 1.02 to 3.20, *p* = .04), the degree of contralateral carotid stenosis, and time to operation of >14 days after the day of randomization (RR 3.33, 95% CI 1.05 to 10.57, *p* = .04).

**Table 2:** Independent predictors of risk of cranial nerve palsy within 30 days of carotid endarterectomy in ICSS per-protocol participants (*n* = 805) in whom the procedure was initiated. Patients with missing data were excluded from this analysis.

Variable	Adjusted risk ratio (95% CI)	Adjusted *p* value
Cardiac failure	2.66 (1.11 to 6.40)	.03
Female sex	1.80 (1.02 to 3.20)	.04
Time from randomization to treatment >14 days	3.33 (1.05 to 10.57)	.04
Degree of contralateral stenosis		Overall *p* < .01
0–50%	1.00	
50–69%	1.18 (0.62 to 2.27)	.62
>70%	0.13 (0.02 to 0.91)	.04

### Haematoma

Of 821 patients in whom the surgical procedure was initiated, 50 (6.1%) developed neck haematoma. Twenty-eight of the 821 (3.4%) were classified as severe.

The results of univariable regression analysis for the risk factors for haematoma development are presented in Appendix II. Statistically significant predictors of increased risk of haematoma were: anticoagulant prescription preoperatively (RR 1.83, 95% CI 1.04 to 3.23, *p* = .04), previous cardiac bypass graft surgery (CABG) (RR 2.46, 95% CI 1.37 to 4.42, *p* < .01), atrial fibrillation (RR 2.29, 95% CI 1.08 to 4.85, *p* = .03), and the duration of arterial clamping in minutes (RR per each extra 20 minutes 1.13, 95% CI 1.04 to 1.24, *p* < .01). Factors associated with a decreased risk of postoperative haematoma were shunt use (RR 0.54, 95% CI 0.29 to 0.99, *p* = .05), antiplatelet agent prescription prior to the procedure (RR 0.44, 95% CI 0.21 to 0.93, *p* = .03) and each 1 mmol/l increase in cholesterol at baseline (RR 0.69, 95% CI 0.55 to 0.88, *p* < .01). Other demographic and technical factors did not predict haematoma.

The results of multivariable analysis of predictors of risk for haematoma are presented in Appendix III. Independent predictors of increased risk were being female (RR 2.03, 95% CI 1.13 to 3.62, *p* = .02), having atrial fibrillation (RR 2.38, 95% CI 1.07 to 5.27, *p* = .03), and the prescription of anticoagulant pre-procedure (RR 1.86, 95% CI 1.01 to 3.42, *p* = .05). Independent factors reducing the risk of haematoma were shunt use (RR 0.40, 95% CI 0.21 to 0.80, *p* < .01) and each 1 mmol/l increase in the patient's baseline cholesterol level (RR 0.68, 95% CI 0.54 to 0.86, *p* < .01).

Twelve of 45 (26.7%) patients with CNP also suffered haematoma, versus 38/776 (4/9%) of patients without CNP. There was a significant association between these complications as detailed in Table 3 (*p* < .01, Fisher's exact test).

**Table 3:** Numbers (%) of patients with haematoma by presence of cranial nerve palsy (CNP)^a^ within 30 days of carotid endarterectomy in ICSS per-protocol participants (n=821).

		Haematoma		
		Number (%)	Yes (%)	Total (%)
CNP	No (%)	738 (95.1)	38 (4.9)	776 (100)
	Yes (%)	33 (73.3)	12 (26.7)	45 (100)
	Total	771	50	821

a *p* < .01 by Fisher's exact test of independence between haematoma and CNP.

### Impact on trial outcomes

Table 4 details the impact of adding CNP to the combined incidence of stroke, myocardial infarction (MI), or death in ICSS in a post-hoc analysis comparing CEA with CAS. There was no significant difference in the combined risk of stroke, MI, death, or CNP, nor was there a significant difference in the incidence of disabling stroke, disabling CNP, or death between the two trial arms.

**Table 4:** Composite outcome events within 30 days of carotid stenting (CAS) versus carotid endarterectomy (CEA) in ICSS, with or without the addition of cranial nerve palsy (CNP), in per-protocol participants.

Endpoint	CAS (*n* = 828) No. events (%)	CEA (*n* = 821) No. events (%)	Risk ratio (95% CI)	Risk difference (95% CI)	*p* (chi-square)
Stroke, MI or death^a^	61 (7.4%)	33 (4.0%)	1.83 (1.21, 2.77)	3.3% (1.1, 5.6)	<.01
Stroke, MI, death or CNP	62 (7.5%)	76 (9.3%)	0.81 (0.59, 1.12)	−1.8% (−4.4, 0.9)	.20

Disabling stroke or death	26 (3.1%)	18 (2.2%)	1.43 (0.79, 2.59)	0.9% (−0.6, 2.5)	.23
Disabling stroke, disabling CNP or death	27 (3.3%)	19 (2.3%)	1.41 (0.79, 2.51)	0.9% (−0.6, 2.5)	.24

a Reported in *Lancet* 2010;376:985–997.

## Discussion

In ICSS, CNP developed in 5.5% of ICSS patients undergoing CEA, and haematoma in 6.1%. There was a statistically significant association between the two outcomes. The largest available series of patients studied pre- and post-operatively, in the European Carotid Surgery Trial (ECST), found a motor CNP rate of 5.1% and a long-term CNP rate of 0.5% at 4 months.^11^ Likewise, NASCET reported an overall risk of postoperative CNP of 8.6%, of which the majority were mild in severity,^12^ suggesting that CNP rates remain constant over time.

Patients should be made aware of these common complications and the likely clinical effects, including sensory and possible motor consequences. They can be reassured from the evidence presented here that postoperative CNP is rarely disabling (risk around 1 in 1000 operations), but should be warned that the symptoms of the CNP may persist for several weeks or longer.

There are several other findings of interest from this study: to the authors' knowledge, the association between female sex and CNP has not previously been described, and this finding is worth confirming in another cohort of patients. One possible explanation for higher risk in female patients may be more challenging surgical anatomy and the smaller average diameter of the carotid artery.^13^ Female patients were also at higher risk of haematoma. Combined with other reports of a higher perioperative stroke risk in symptomatic women undergoing CEA versus men,^14^ and evidence that the net benefit of CEA in women is lower than in men,^15^ an increased incidence of CNP should be borne in mind when advising female patients of their risk of complications following CEA.

Other risk factors for CNP identified in this analysis, including the degree of contralateral carotid stenosis, are statistically significant predictors but are more difficult to link clinically with the outcome and have not, to the authors' knowledge, been reported by other groups.

The association of neck haematoma with pre-operative anticoagulation is not surprising, but emphasizes the importance of careful surgical technique to mitigate the risk of haematoma in anticoagulated patients. A risk of haematoma in ICSS of 6.1% is similar to the rate of “wound complications” seen in large case series^16^ and a severe haematoma risk of 3.4% is similar to the reported risk of re-exploration of the surgical wound required in patients undergoing CEA while on antiplatelet medication.^17^ However, although there is concern about perioperative bleeding in patients on dual antiplatelet therapy, the incidence of haematoma was actually decreased in this study in patients taking these medications, perhaps because surgeons took more care with haemostasis in these patients.

Some systematic reviews have included CNP in a composite outcome event of death or neurological complications up to 30 days after treatment.^18^ ICSS compared CAS with CEA on the assumption that if CAS could avoid neck incision, CNP, and haematoma with no excess risk of stroke, then it could provide a beneficial alternative to CEA for the prevention of recurrent stroke in patients with symptomatic carotid stenosis.^10^ In ICSS, as reported here, the total number of events in the composite cluster of any stroke, MI, death, or CNP was greater after CEA compared with CAS, but the numbers in the cluster of disabling stroke, disabling CNP or death were greater after CAS than after CEA. Neither difference was statistically significant. It is concluded, therefore, that it would not be appropriate to base treatment considerations concerning the choice of CAS versus CEA on the basis of composite short-term endpoints including CNP.

### Limitations of the analysis

The present analysis has some important limitations. In some patients, information regarding baseline risk factors was unavailable, and information about the duration of CNP symptoms was limited. Multiple comparisons without statistical correction raise the possibility of obtaining a Type I (false positive) error. Results could be confirmed in other patient cohorts. This is not a randomized comparison of surgical techniques or perioperative processes of care, and it is possible that unmeasured confounders are associated with the risk of CNP or haematoma.

### Conclusion

CNP remains a relatively common complication of CEA, but in many patients is transient. Haematoma is similarly common, and there is a statistical association between haematoma and CNP. Women should be warned about an increased risk of CNP. Scrupulous attention to haemostasis might reduce the incidence of CNP. Fortunately, prolonged disability or permanent symptoms as a result of haematoma or CNP are rare, and thus, in the authors' opinion, do not warrant inclusion in composite endpoints for future trials of carotid revascularization, but nevertheless one in 821 CEA patients in ICSS had permanent impairment of swallowing caused by cranial nerve palsy.
