# Supplementary material for: Incidence, Impact, and Predictors of Cranial Nerve Palsy and Haematoma Following Carotid Endarterectomy in the International Carotid Stenting Study
Source: Eur J Vasc Endovasc Surg. 2014 Nov;48(5):498–504. doi: 10.1016/j.ejvs.2014.08.002 (PMC4225222; doi:10.1016/j.ejvs.2014.08.002)
Supplement: Supplementary file 1 [file mmc1.doc]

**ICSS Cranial Nerve Palsy Questionnaire**

| Centre ID: |  |  |  |  | | | | | | | | | | | | | | | |
| --- | --- | --- | --- | --- | --- | --- | --- | --- | --- | --- | --- | --- | --- | --- | --- | --- | --- | --- | --- |
| ICSS trial number: |  |  |  |  | | |  | | | | | | | | | | | | |
| Patient date of birth (dd/mm/yyyy): |  |  |  |  | | |  | |  | |  | |  | | | |  | | |
|  |  | | | | | | | | | | | | | | | | | | |
| Date of carotid endarterectomy (dd/mm/yyyy): |  |  |  |  | | |  | |  | |  | |  | | | |  | | |
| Date of cranial nerve palsy (dd/mm/yyyy): |  |  |  |  | | |  | |  | |  | |  | | | |  | | |
| Date of completion of this questionnaire  (dd/mm/yyyy): |  |  |  |  | | |  | |  | |  | |  | | | |  | | |
| Name of person completing this questionnaire: |  | | | | | | | | | | | | | | | | | | |
|  |  | | | | | | | | | | | | | | | | | | |
| Which cranial nerves were affected? (*delete as appropriate)* | Vagal | | | | | | | | | Yes / No | | | | | | | | | |
|  | Hypoglossal | | | | | | | | | Yes / No | | | | | | | | | |
|  | Glossopharyngeal | | | | | | | | | Yes / No | | | | | | | | | |
|  | Accessory | | | | | | | | | Yes / No | | | | | | | | | |
|  | Facial | | | | | | | | | Yes / No | | | | | | | | | |
|  |  | | | | | | | | | | | | | | | | | | |
| Are details of the cranial nerve palsy documented in the clinical notes? (*delete as appropriate*) ***** | Yes / No | | | | | | | | | | | | | | | | | | |
| What symptoms were recorded as a result of cranial nerve palsy? (*see aide memoire for suggestions)* |  | | | | | | | | | | | | | | | | | | |
| What neurological signs were recorded as a result of cranial nerve palsy? (*see aide memoire for suggestions)* |  | | | | | | | | | | | | | | | | | | |
| What functional consequences occurred as a result of the cranial nerve palsy (*e.g. patient had to have a nasogastric tube*) |  | | | | | | | | | | | | | | | | | | |
| How long did the cranial nerve palsy last? ***** |  |  | days | | |  | |  | | weeks | | | | |  | | |  | months |
| Was hospital discharge delayed by cranial nerve palsy? (*delete as appropriate*)  If so, please estimate by how long: | Yes / No | | | | | | | | | | | | | | | | | | |
|  |  |  | days | |  | | |  | | weeks | | | |  | | | |  | months |
| Did the patient receive investigation for cranial nerve palsy? (*delete as appropriate*)  If yes, what investigation?  What date was this carried out? (dd/mm/yyyy): | Yes / No | | | | | | | | | | | | | | | | | | |
|  |  |  |  |  |  |  |  |  |  |  |  |  |  |  |  |  |  |  |  |
|  |  |  |  |  |  |  |  |  |  |  |  |  |  |  |  |  |  |  |  |
| Did the patient receive treatment for cranial nerve palsy?  If yes, what treatment?  What date was this carried out? (dd/mm/yyyy): | Yes / No | | | | | | | | | | | | | | | | | | |
|  |  |  |  |  |  |  |  |  |  |  |  |  |  |  |  |  |  |  |  |
|  |  |  |  |  |  |  |  |  |  |  |  |  |  |  |  |  |  |  |  |

*** If CNP not documented or date of resolution is unknown please contact the patient, if alive, to see if they can answer the questions that follow**

**ICSS Cranial Nerve Palsy - *Aide Memoire***

Clinical symptoms of cranial nerve palsy

| Vagal | Change in voice / hoarseness of voice |
| --- | --- |
|  | Difficulty swallowing |
|  | Choking / cough |
|  | |
| Hypoglossal | Problems with tongue / clumsiness |
|  | Difficulty swallowing |
|  | |
| Glossopharyngeal | Difficulty swallowing |
|  | |
| Accessory | Shoulder droop |
|  | Winged scapula |
|  | |
| Facial | Uplifting of the mouth on one side |
|  | Facial droop |

| Any other comments regarding this patient’s cranial nerve palsy? |
| --- |
|  |
