# Supplementary material for: Incidence, Impact, and Predictors of Cranial Nerve Palsy and Haematoma Following Carotid Endarterectomy in the International Carotid Stenting Study
Source: Eur J Vasc Endovasc Surg. 2014 Nov;48(5):498–504. doi: 10.1016/j.ejvs.2014.08.002 (PMC4225222; doi:10.1016/j.ejvs.2014.08.002)
Supplement: Supplementary file 2 [file mmc2.doc]

**Appendix II.** Univariable predictors of risk of haematoma within 30 days of endarterectomy in 821 ICSS per-protocol participants in whom the procedure was initiated

| **Variable** | **Category** | **Number at risk** | **Number of events** | **30-day rate of haematoma (%)** | **Risk ratio (95% CI)** | ***p* value** |
| --- | --- | --- | --- | --- | --- | --- |
| **Technical factors** | | | | | | |
| Type of reconstruction | Standard | 182 | 17 | 9.3 | 1 | .26 |
|  | Eversion | 49 | 4 | 8.2 | 0.87 (0.31 to 2.48) |  |
|  | Patch | 459 | 24 | 5.2 | 0.56 (0.31 to 1.02) |  |
|  | Vein interposition | 3 | 0 | 0 | N/A |  |
|  | Data missing | 128 | 5 |  | | |
| Shunt use | No | 494 | 37 | 7.5 | 1 | .05 |
|  | Yes | 324 | 13 | 4.0 | 0.54 (0.29 to 0.99) |  |
|  | Data missing | 3 | 0 |  | | |
| Type of anaesthesia | GA or LA/GA combined | 650 | 42 | 6.5 | 1 | .69 |
|  | LA alone | 144 | 8 | 5.6 | 0.86 (0.41 to 1.79) |  |
|  | Data missing | 27 | 0 |  | | |
| Antiplatelet agent pre-procedure | No | 52 | 7 | 13.5 | 1 | .03 |
|  | Yes | 726 | 43 | 5.9 | 0.44 (0.21 to 0.93) |  |
|  | Data missing | 43 | 0 |  | | |
| Two or more antiplatelet agents pre-procedure | No | 531 | 32 | 6.0 | 1 | .50 |
|  | Yes | 247 | 18 | 7.3 | 1.21 (0.69 to 2.11) |  |
|  | Data missing | 43 | 0 |  | | |
| Anticoagulant pre-procedure | No | 619 | 34 | 5.5 | 1 | .04 |
|  | Yes | 159 | 16 | 10.1 | 1.83 (1.04 to 3.23) |  |
|  | Data missing | 43 | 0 |  | | |
| Antiplatelet agent post-procedure | No | 87 | 8 | 9.2 | 1 | .26 |
|  | Yes | 691 | 42 | 6.1 | 0.66 (0.32 to 1.36) |  |
|  | Data missing | 43 | 0 |  | | |
| Anticoagulant post-procedure | No | 574 | 32 | 5.6 | 1 | .11 |
|  | Yes | 204 | 18 | 8.8 | 1.58 (0.91 to 2.76) |  |
|  | Data missing | 43 | 0 |  | | |
| **Patient factors** | | | | | | |
| Sex | Male | 578 | 30 | 5.2 | 1 | .10 |
|  | Female | 243 | 20 | 8.2 | 1.59 (0.92 to 2.74) |  |
|  | Data missing | 0 | 0 |  | | |
| Age | <70 years | 391 | 24 | 6.1 | 1 | .96 |
|  | ≥70 years | 430 | 26 | 6.0 | 0.99 (0.58 to 1.69) |  |
|  | Data missing | 0 | 0 |  | | |
| Treated hypertension | No | 242 | 12 | 5.0 | 1 | .36 |
|  | Yes | 572 | 38 | 6.6 | 1.34 (0.71 to 2.52) |  |
|  | Data missing | 7 | 0 |  | | |
| Cardiac failure | No | 770 | 47 | 6.1 | 1 | .85 |
|  | Yes | 44 | 3 | 6.8 | 1.12 (0.36 to 3.45) |  |
|  | Data missing | 7 | 0 |  | | |
| Angina | No | 742 | 45 | 6.1 | 1 | .77 |
|  | Yes | 72 | 5 | 6.9 | 1.15 (0.47 to 2.79) |  |
|  | Data missing | 7 | 0 |  | | |
| Previous MI | No | 664 | 36 | 5.4 | 1 | .07 |
|  | Yes | 150 | 14 | 9.3 | 1.72 (0.95 to 3.11) |  |
|  | Data missing | 7 | 0 |  | | |
| Previous CABG | No | 703 | 36 | 5.1 | 1 | <.01 |
|  | Yes | 111 | 14 | 12.6 | 2.46 (1.37 to 4.42) |  |
|  | Data missing | 7 | 0 |  | | |
| Atrial fibrillation | No | 760 | 43 | 5.7 | 1 | .03 |
|  | Yes | 54 | 7 | 13.0 | 2.29 (1.08 to 4.85) |  |
|  | Data missing | 7 | 0 |  | | |
| Other cardioembolic source of embolus | No | 798 | 49 | 6.1 | 1 | .99 |
|  | Yes | 16 | 1 | 6.3 | 1.02 (0.15 to 6.92) |  |
|  | Data missing | 7 | 0 |  | | |
| Diabetes | No | 640 | 40 | 6.3 | 1 | .81 |
|  | Yes | 174 | 10 | 5.7 | 0.92 (0.47 to 1.80) |  |
|  | Data missing | 7 | 0 |  | | |
| Peripheral arterial disease | No | 683 | 43 | 6.3 | 1 | .68 |
|  | Yes | 131 | 7 | 5.3 | 0.85 (0.39 to 1.85) |  |
|  | Data missing | 7 | 0 |  | | |
| Smoking status | Never smoked | 220 | 9 | 4.1 | 1 | .23 |
|  | Former smoker | 404 | 30 | 7.4 | 1.82 (0.88 to 3.75) |  |
|  | Current smoker | 190 | 11 | 5.8 | 1.42 (0.60 to 3.34) |  |
|  | Data missing | 7 | 0 |  | | |
| Treated hyperlipidaemia | No | 275 | 18 | 6.5 | 1 | .73 |
|  | Yes | 539 | 32 | 5.9 | 0.91 (0.52 to 1.59) |  |
|  | Data missing | 7 | 0 |  | | |
| Degree of stenosis in treated artery | 50–69% | 72 | 5 | 6.9 | 1 | .75 |
|  | 70–99% | 749 | 45 | 6.0 | 0.87 (0.36 to 2.11) |  |
|  | Data missing | 0 | 0 |  | | |
| Degree of stenosis in contralateral artery | 0‑49% | 539 | 33 | 6.1 | 1 | .39 |
|  | 50‑69% | 136 | 10 | 7.4 | 1.20 (0.61 to 2.38) |  |
|  | 70‑99% | 104 | 3 | 2.9 | 0.47 (0.15 to 1.51) |  |
|  | Occluded | 35 | 3 | 8.6 | 1.40 (0.45 to 4.34) |  |
|  | Data missing | 7 | 1 |  | | |
| Nature of ipsilateral index event | Stroke | 360 | 18 | 5 | 1 | .32 |
|  | Retinal stroke | 22 | 1 | 4.5 | 0.91 (0.13 to 6.51) |  |
|  | TIA | 292 | 18 | 6.2 | 1.23 (0.65 to 2.33) |  |
|  | Amaurosis fugax | 134 | 13 | 9.7 | 1.94 (0.98 to 3.85) |  |
|  | Data missing | 13 | 0 |  | | |
| Multiple ipsilateral events prior to randomization | No | 513 | 28 | 5.5 | 1 | .33 |
|  | Yes | 308 | 22 | 7.1 | 1.31 (0.76 to 2.25) |  |
|  | Data missing | 0 | 0 |  | | |
| Prior ipsilateral stroke | No | 718 | 42 | 5.8 | 1 | .45 |
|  | Yes | 103 | 8 | 7.8 | 1.33 (0.64 to 2.75) |  |
|  | Data missing | 0 | 0 |  | | |
| Baseline Rankin score | 0 | 307 | 20 | 6.5 | 1 | .42 |
|  | 1 | 194 | 12 | 6.2 | 0.95 (0.48 to 1.90) |  |
|  | 2 | 212 | 15 | 7.1 | 1.09 (0.57 to 2.07) |  |
|  | 3 | 74 | 1 | 1.4 | 0.21 (0.03 to 1.52) |  |
|  | 4 | 17 | 1 | 5.9 | 0.90 (0.13 to 6.33) |  |
|  | 5 | 3 | 0 | 0 | N/A |  |
|  | Data missing | 0 | 0 |  | | |
| Side of procedure | Left | 427 | 25 | 5.9 | 1 | .77 |
|  | Right | 394 | 25 | 6.3 | 1.08 (0.63 to 1.86) |  |
|  | Data missing | 0 | 0 |  | | |
| Time from randomization to treatment | ≤14 days | 151 | 7 | 4.6 | 1 | .41 |
|  | >14 days | 668 | 43 | 6.4 | 1.39 (0.64 to 3.03) |  |
|  | Data missing | 2 | 0 |  | | |
| **Continuous variables** | | | | | | |
| Clamping time | (Per 20 minutes of clamp applied) | 609 | 42 | 6.9 | 1.13 (1.04 to 1.24) | <.01 |
|  | Data missing | 212 | 8 |  | | |
| Duration of surgery | (Per 20 minutes of operation length) | 685 | 44 | 6.4 | 0.97 (0.86 to 1.10) | .66 |
|  | Data missing | 136 | 6 |  | | |
| Age | (Per 5 years of age) | 821 | 50 | 6.1 | 1.02 (0.88 to 1.18) | .84 |
|  | Data missing | 0 | 0 |  | | |
| Baseline systolic blood pressure | (Per 10mmHg of blood pressure) | 784 | 49 | 6.3 | 1.08 (0.98 to 1.21) | .13 |
|  | Data missing | 37 | 1 |  | | |
| Baseline diastolic blood pressure | (Per 10mmHg of blood pressure) | 785 | 49 | 6.2 | 0.92 (0.74 to 1.14) | .45 |
|  | Data missing | 36 | 1 |  | | |
| Baseline cholesterol | (Per each mmol/l total cholesterol) | 679 | 43 | 6.3 | 0.69 (0.55 to 0.88) | <.01 |
|  | Data missing | 142 | 7 |  | | |
| Time from index event to procedure | (Per 7 days) | 819 | 50 | 6.1 | 1.00 (0.97 to 1.02) | .73 |
|  | Data missing | 2 | 0 |  | | |
