# Supplementary material for: Incidence, Impact, and Predictors of Cranial Nerve Palsy and Haematoma Following Carotid Endarterectomy in the International Carotid Stenting Study
Source: Eur J Vasc Endovasc Surg. 2014 Nov;48(5):498–504. doi: 10.1016/j.ejvs.2014.08.002 (PMC4225222; doi:10.1016/j.ejvs.2014.08.002)
Supplement: Supplementary file 3 [file mmc3.doc]

**Appendix III.** Independent predictors of the risk of haematoma within 30 days of carotid endarterectomy in 639^a^ ICSS per-protocol participants in whom the procedure was initiated

| **Variable** | **Adjusted risk ratio (95% CI)** | **Adjusted *p* value** |
| --- | --- | --- |
| Anticoagulant pre-procedure | 1.86 (1.01 to 3.42) | .05 |
| Shunt use | 0.40 (0.21 to 0.80) | <.01 |
| Cholesterol (per each mmol/l) | 0.68 (0.54 to 0.86) | <.01 |
| Female | 2.03 (1.13 to 3.62) | .02 |
| Atrial fibrillation | 2.38 (1.07 to 5.27) | .03 |

^a^ Patients with missing data excluded from this analysis.
